# Editorial comment: Renal denervation

**DOI:** 10.1038/s41440-021-00808-w

**Published:** 2021-12-16

**Authors:** Roland E. Schmieder, Agnes Bosch

**Affiliations:** Department of Nephrology and Hypertension, University Hospital Erlangen, Friedrich-Alexander-University Erlangen-Nürnberg (FAU), Erlangen, Germany

Several recently published randomised sham-controlled trials have demonstrated significant blood pressure (BP) reductions following renal denervation (RDN) in patients with hypertension, both in the presence and absence of antihypertensive therapy [1]. The REQUIRE trial by Kario et al. [2] is the first trial of ultrasound renal denervation in Asian patients with hypertension receiving antihypertensive drug therapy, adds important value to our understanding of RDN induced BP reduction, and invites readers to have a closer look at confounding factors in RDN trials.

In the REQUIRE trial both the treatment and sham groups showed a significant reduction in 24 h ambulatory systolic BP 3 months after RDN: −6.6 mmHg in the RDN and −6.5 mmHg in the sham control group [2]. The study findings were neutral for the primary endpoint, with similar BP reductions in the two groups. Although BP reduction in the RDN was similar to other sham-controlled studies [3–5], the sham control group in this study showed a much greater reduction than in comparable clinical trials using the same or other catheter systems [5]. How can we explain these results? The ultrasound catheter technique used in the REQUIRE trial has been previously shown to be safe and effective [3, 4]. Similarly, there was no signal indicating a safety issue in the REQUIRE study [2]. Considering patient selection and inclusion criteria of the REQUIRE trial, it is important to note that patients with presumed hyperaldosteronism have been included. Forty-four out of 142 patients in the total trial population and 18 out of 72 patients in the treatment group had hyperaldosteronism as indicated by aldosterone/renin ratios >200 [2]. In a post-hoc analysis excluding these 44 patients, the reduction in 24 h ambulatory systolic BP from baseline at 3 month was somewhat higher in the RDN group (−7.6 mmHg instead of −6.6 mmHg) [2]. Patients with presumed hyperaldosteronism have been found to have a reduced sympathetic nerve activity and are therefore very likely to poorly respond to RDN [6]. This might explain why the reduction in 24 h systolic BP in the RDN group was −6.6 mmHg in the REQIRE trial, whereas other clinical trial using the same RDN device showed −7.0 mmHg (RADIANCE-HTN SOLO) [3] and −8.5 mmHg (RADIANCE-HTN TRIO) [4]. The standard deviation of 16.1 mmHg in the REQUIRE trial for change of 24 h ambulatory systolic BP in the RDN group at 3 months is higher than observed in other similar trials (e.g. 10 mmHg in RADIANCE-HTN SOLO) [3]. This high standard deviation might be related to the inclusion of patients with hyperaldosteronism and the high number of study centres (72 centres included 142 patients). In the SYMPLICITY HTN-3 study it was discussed that a high number of RDN centres increases the variability of the data.

The second astonishing finding of the REQUIRE study is the high BP decrease in the sham control group (−6.5 mmHg in 24 h ambulatory systolic BP) [2]. In the RADIANCE-HTN SOLO [3] and TRIO [4] studies the magnitude of reductions in 24 h ambulatory SBP from baseline in the sham control group were much lower (−3.1 mmHg and −2.9 mmHg, respectively). As nicely evaluated in the discussion section of the REQUIRE study, medication adherence might be the key explanation for the exaggerated BP response in the sham control group [2]. Doctors as well as nurses treating the patients included in the REQUIRE trial have not been blinded thereby creating a substantial bias. Blinding index was neither evaluated in patients nor medical professionals. Another important point is that changes in antihypertensive medication during the first 3 months after RDN have been reported by the patient, but not been validated by urinary toxicological drug measurements of antihypertensive drugs [7]. Thus, there was no true control of patient medication adherence in both the RDN and the sham control group during the first 3 months after RDN.

Published in the same issue of Hypertension Research, a systematic review and meta-analysis of RDN randomised sham-controlled trials concluded that RDN significantly reduced all BP metrics, both in the presence and absence of concomitant antihypertensive pharmacotherapy [8]. The systematic review provides a very interesting insight in the heterogeneity of the integrated clinical trials and invites us to have a closer look at the reasons why some trials were not successful in terms of RDN superiority compared to medical treatment. Interestingly, first as well as second generation RDN clinical trials have been included in the meta-analysis [8], regardless the fact that RDN technology and methodology and conductance of trials improved substantially compared to the 1st generation trials. The meta-analysis by Oguyama et al. nicely visualises this superiority of 2nd generation trials compared to 1st generation trials in its summarising graphical illustration. Whereas the SYMPLICITY HTN-3 study had several major methodological problems and used technique dependent older technologies, which has been nicely evaluated by Kandzari et al. [1], the SYMPLICITY-FLEX study (that also used the 1st generation radiofrequency catheter) utilised a different study design, and the REDUCE HTN: REINFORCE trial was stopped early, prohibiting a conclusive analysis. It was these trials that showed a lower response in 24 h ambulatory systolic BP compared to the 2nd generation trials. Thus, also including studies with poor methodology and technology, in particular the SYMPLICITY HTN-3 trial [1] (and other 1st generation trials), the magnitude of the BP decrease after RDN with contemporary technologies cannot be precisely derived from such a meta-analysis. The second clinical consensus conference on device based hypertension therapies [7] selected only high quality 2nd generation trials and the position paper on RDN of the European Society of Hypertension emphasises this by stating that 2nd generation clinical trials with improved technology, trial design and patient selection provide conclusive evidence that RDN lowers ambulatory and office BP to a significantly greater extent than sham treatment [5]. For further analysis we recommend to focus only on second generation RDN clinical trials.

Finally, the “Japan nationwide web-based survey of patient preference for RDN for hypertension treatment” by Kario et al. [9] adds important information in our understanding of the patient’s view on RDN. This study evaluated patient preference for RDN in patients with hypertension from Japan [9]. A total of almost 2400 patients were included with one-third expressing preference for RDN. Significant predictors of preference for RDN were younger patient age, male sex, higher home or office systolic BP, poor antihypertensive drug adherence, the presence of heart failure, and the presence of side effects during treatment with antihypertensive drugs [9]. The results of this study help to encourage the shared decision-making process between patients and medical professionals about antihypertensive therapy, and therefore provides an important insight of patients’ perspective which needs to be respected when implementing RDN as an innovative option in the armamentarium of antihypertensive treatment.
